# Current Management of Aneurysmal Subarachnoid Hemorrhage

**DOI:** 10.3390/neurolint17030036

**Published:** 2025-02-26

**Authors:** Jay Max Findlay

**Affiliations:** Division of Neurosurgery, Department of Surgery, University of Alberta, 2D1.02 Mackenzie Health Sciences Centre, 8440-112 Street, Edmonton, AB T6G 2B7, Canada; jmaxfindlay@gmail.com; Tel.: +1-780-407-3548

**Keywords:** subarachnoid hemorrhage, cerebral aneurysm, cerebral vasospasm, acute hydrocephalus

## Abstract

The diagnosis of aneurysmal subarachnoid hemorrhage (aSAH) is most difficult in patients who are in good clinical condition with a small hemorrhage, especially when a ruptured aneurysm might not be considered, or if a computed tomographic (CT) scan is not obtained, or if when a CT is obtained, the findings are subtle and missed by an inexperienced reviewer. All acute onset (thunderclap) headaches should be considered ruptured aneurysms until proven otherwise. Treatment begins with immediate control of pain and blood pressure, placement of an external ventricular drain (EVD) in poor-grade patients and those with acute hydrocephalus on CT scanning, administration of antifibrinolytic tranexamic acid, and then repair of the aneurysm with either surgical clipping or endovascular techniques as soon as the appropriate treatment team can be assembled. After securing the aneurysm, aSAH patient treatment is focused on maintaining euvolemia and a favorable systemic metabolic state for brain repair. A significant and aneurysm-specific threat after aSAH is delayed arterial vasospasm and resulting cerebral ischemia, which is detected by vigilant bedside examinations for new-onset focal deficits or neurological decline, assisted with daily transcranial Doppler examinations and the judicious use of vascular imaging and cerebral perfusion studies with CT. The management of diagnosed symptomatic vasospasm is the prompt induction of hypertension with vasopressors, but if this fails to reverse deficits quickly after reaching a target systolic blood pressure of 200 mmHg, endovascular angioplasty is indicated, providing CT scanning rules out an established cerebral infarction. Balloon angioplasty should be considered early for all patients found to have severe angiographic vasospasm, with or without detectable signs of ischemic neurological deterioration due to either sedation or a pre-existing deficit.

## 1. Introduction

Spontaneous subarachnoid hemorrhage (SAH) refers to intracranial bleeding into the cerebrospinal-filled space beneath the arachnoid membrane covering the brain, not associated with any form of trauma. The most common single cause is the rupture of a saccular intracranial aneurysm arising from one of the larger conducting arteries traveling through the subarachnoid space at the base of the brain. Saccular intracranial aneurysms are acquired lesions, “developmental” in etiology, usually forming in adulthood at branch points and bifurcations of larger cerebral arteries, possibly related to a weakness in the arterial wall subject to prolonged hemodynamic stress [1]. Ruptured aneurysms, which account for roughly 10% of all cerebrovascular accidents [2], present with a thunderclap headache, nausea and vomiting, and usually a decreased level of consciousness, sometimes coma. As it is a high-pressure and often high-volume arterial hemorrhage, aneurysmal subarachnoid hemorrhage (aSAH) is associated with high early mortality and significant long-term morbidity. But as we will outline below, prompt and collaborative multidisciplinary care from emergency, intensive care, neurosurgical, and neuroradiology interventional services can save lives and optimize outcomes in SAH survivors.

Trauma and indeed any cause of intracerebral bleeding can be associated with SAH, providing the hematoma reaches the pial surface of the brain, examples being vascular malformations, brain tumors, amyloid angiopathy, and bleeding disorders, none of which will be covered in this chapter. However, in these patients, the volume of subarachnoid blood is generally localized and limited, so the associated SAH is not a major consideration in patient management or outcomes.

There are several less common types of spontaneous SAHs that closely resemble aSAH in terms of presentation and clinical behavior. The most common of these, accounting for roughly 20% of spontaneous SAHs, is known as “peri mesencephalic”, “pre trunkal”, or “non-aneurysmal”, SAH where no aneurysm is found, and the smaller volume hemorrhage is in the subarachnoid space around the upper brainstem (“mesencephalon” refers to the midbrain) [3]. Much less common are arteriovenous dural fistulae, where draining veins bridge the subarachnoid space, and spontaneous intracranial arterial dissections. While rare, these three conditions are always in the differential diagnosis of an aSAH and so will be touched on in our discussion.

## 2. Clinical Aspects

The typical aSAH patient is middle aged, and women slightly outnumber men. Risk factors for saccular aneurysm formation and/or rupture include hypertension, cigarette smoking, severe alcoholism, sympathomimetic drug use such as cocaine, having had a prior aSAH and a strong family history of cerebral aneurysms (two or more first-degree relatives) [4]. Some heritable conditions are associated with intracranial aneurysms, but the clinically relevant one is autosomal dominant polycystic disease, where up to 10% of patients will develop a saccular aneurysm. Other sometimes-mentioned connective tissue disorders, such as Ehlers-Danlos syndrome and neurofibromatosis, can predispose to the formation of cerebral artery ectasias and “fusiform” intracranial aneurysms.

Aneurysms are found in between 2 and 4% of people worldwide, with the incidence higher in certain populations, Japan and Finland, for example [5]. When discovered unruptured, risk factors for future rupture must be considered, including a history of rupture from another, treated aneurysm, larger aneurysm diameter or an irregular shape, more dangerous aneurysm locations (such as the posterior circulation arteries), and a family history of cerebral aneurysms [6]. The average risk of rupture of an incidentally discovered saccular aneurysm is 1 or 2% per year, perhaps 3% for a “high risk” aneurysm [7], so treatment recommendations are based on lifetime “cumulative risk” tables (Table 1), that risk far higher in younger patients. A higher-risk aneurysm in a younger patient is considered for prophylactic repair, but many incidentally discovered small aneurysms (<5 mm) are simply followed, especially over the age of 60.

While certain events can coincide with aSAH, including exercise, intercourse, and cocaine use, nothing is considered a clear-cut trigger, and patients with unruptured aneurysms are advised to live their lives without activity restrictions.

Aneurysm rupture can cause parenchymal, intraventricular, and subdural hematomas in addition to aSAH, depending on the aneurysm location and severity of bleeding. All are naturally associated with a more severe clinical presentation and worse prognosis, as will be discussed below. The most immediate risk of a newly diagnosed aSAH is acute aneurysm rebleeding, often fatal, and this will be discussed further in terms of acute management.

## 3. SAH Classifications

The clinical severity of aSAH is classified or “graded” based on the patient’s symptoms and signs. The original grading system was introduced by Botterell and Lougheed at the University of Toronto in the 1950s, a significant advance as it standardized assessment criteria and allowed a way of comparing outcomes in clinically similar groups of patients according to their neurological condition or grade [8]. Soon after, the Americans Hunt and Hess introduced a virtually identical grading system [9], but more widely adopted and still in use today, now complimented by an even more recent World Federation of Neurosurgical Societies (WFNS) scoring system [10]. Along with the generic Glasgow Coma Score, (GCS) but specifically for aSAH, these grading systems help predict clinical outcomes (Table 2).

We will discuss acute hydrocephalus complicating aSAH below, but it is important to note that the clinical grade of a patient should be assigned only after the insertion of an external ventricular drain (EVD) in poor-grade patients and/or those with acute hydrocephalus An EVD placement in these patients often results in rapid clinical improvement and therefore a better grade and prognosis.

The other commonly used classification system measures the severity of bleeding based on CT scanning, the Fisher Scale (the original “modified” by including intraventricular bleeding) [11]. This system assesses the amount of blood in the subarachnoid space as well as any associated intraventricular hematomas (IVHs) and is used to help predict vasospasm, meaning post-SAH large artery narrowing causing symptomatic ischemia, a subject to be discussed later in this review. The thicker and more widespread the hematomas (higher Fisher grades), the higher the risk of symptomatic vasospasm, the risk varying between roughly 20 and 40%. It is worth noting that the predictability is greater when thick hematomas persist for several days on CT scanning since, on occasion, a dense SAH can show considerable clearance over 24 h, corresponding to a reduced vasospasm risk.

## 4. Clinical Presentation

The acute, sudden onset or “thunderclap” headache associated with aSAH, accompanied by nausea, vomiting, and altered consciousness, has been mentioned, and patients can develop meningismus (neck stiffness) in the hours following due to chemical meningitis. Formerly we referred to small aSAHs that remained undiagnosed followed by major rebleeding as “sentinel hemorrhages”, but they are more appropriately thought of as low volume hemorrhages where the patient remained in good condition (good grade), and they either did not seek medical attention, or they did, but their condition was not recognized a serious mistake.

Patients with severe aSAHs causing an altered level of consciousness always undergo CT scanning, which is markedly abnormal, so an aneurysm diagnosis is always made. Patients at greatest risk of being “missed” are those with small hemorrhages, so they remain alert, and therefore, the diagnosis is either not entertained and a CT scan not obtained, or if a CT scan is performed, a subtle SAH might not be recognized by a non-expert.

Because of the grave risk of rebleeding, our rule and teaching is that all sudden-onset headaches should be considered aSAHs until proven otherwise, including those patients with a history of chronic migraine. “Exertional”, including post-coital headaches, are thunderclap in onset, more common in young adults, and patients are characteristically well with a rapidly resolving headache and normal plain CT head. But again, a CT angiogram is warranted to rule out the presence of an aneurysm.

## 5. Diagnosis

At this time, state-of-the-art computed tomography (CT) can detect 100% of aSAHs when performed within hours of presentation and read by the “practiced eye” of an experienced clinician paying particular attention to the basal subarachnoid cisterns [12]. However, if a CT scan has been performed because of a concern of an aneurysm rupture within days following a suspicious headache and is considered normal, additional testing must follow. Increasingly this is CT-angiography looking for the presence of an aneurysm [13]. Formerly, a lumbar puncture (LP) looking for evidence of bleeding in the cerebrospinal fluid (CSF) was recommended, but a painful LP can induce aneurysm rebleeding, and a “traumatic tap”, which is bleeding into the CSF from the LP itself, is a frequent problem, complicating the diagnosis [14].

If an LP is performed, it should be performed by a physician with the knowledge required to distinguish a true from traumatic SAH, and the preferred method is examining the CSF for xanthochromia (visible CSF discoloration or “staining”) following centrifugation (performed by the hospital laboratory, and without spectrophotometry). In the acute setting, the necessary several hours required for intact RBCs to lyse and release hemoglobin into the CSF (which remains in solution after centrifugation) will have passed after patients make their way to the emergency department, their examination, CT scanning performed, and the scans interpreted. It is a useful clue to remember that “traumatic taps” are generally associated with hundreds rather than the thousands or tens of thousands of red blood cells (RBCs) per microliters of CSF seen after a true aSAH.

**Figure 1:**
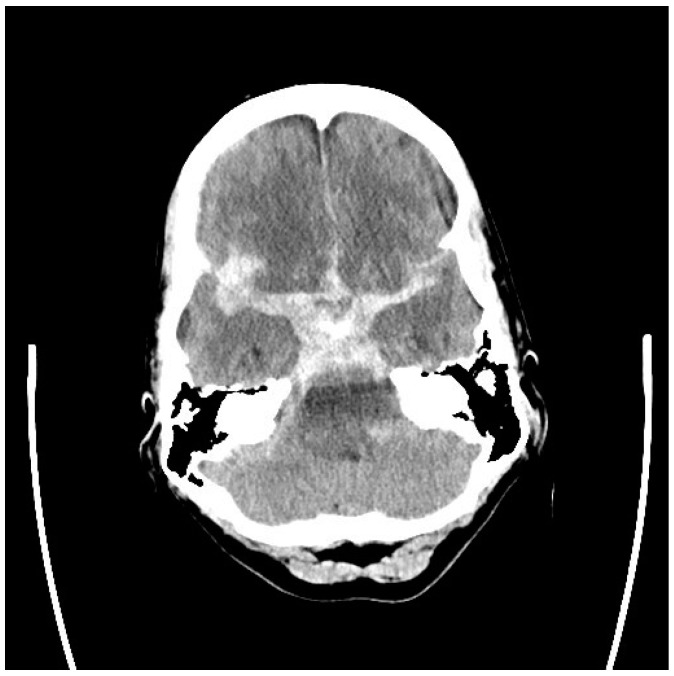
CT scan through the basal subarachnoid cisterns showing thick, hyperdense (white) clots in a 52-year-old woman who was drowsy and confused after an acute onset headache (Hunt and Hess and WFNS grade III). Note the thicker clot in the right Sylvian and insular cisterns (left side of the figure).

**Figure 2:**
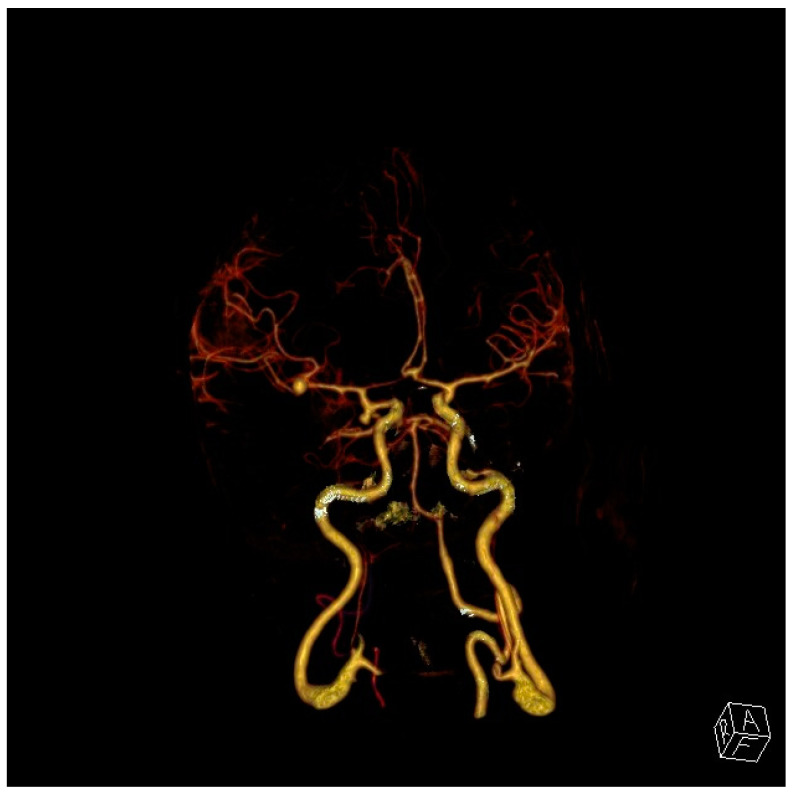
CT angiogram of the same patient shows two right-sided saccular aneurysms (left side of figure), the larger at the right MCA bifurcation and the second arising from the right ICA (up to 20% of aSAH patients have more than one aneurysm). Given that the MCA aneurysm was larger and associated with thicker Sylvian and insular clots, we could be confident it was the source of the bleeding, but both aneurysms were repaired with microsurgical clipping on the day of admission.

Very severe and diffuse aSAHs conceal the source of the bleeding, but more localized hemorrhages will indicate the ruptured aneurysm location, something very important in the management of patients with multiple cerebral aneurysms, found in up to 20% of patients [15] (Figure 1 and Figure 2). Anterior communicating aneurysms (ACommAs) result in basal interhemispheric hematomas, middle cerebral aneurysms (MCAAs) cause Sylvian and insular cistern hematomas, and posterior communicating artery aneurysms (PCommAs) leave carotid cistern hematomas. The location of any associated parenchymal hematoma will also localize the bleeding source. For example, inferior frontal hematomas point to an ACommA aneurysm, and a peri-Sylvian hematoma to an MCAA.

**Figure 3:**
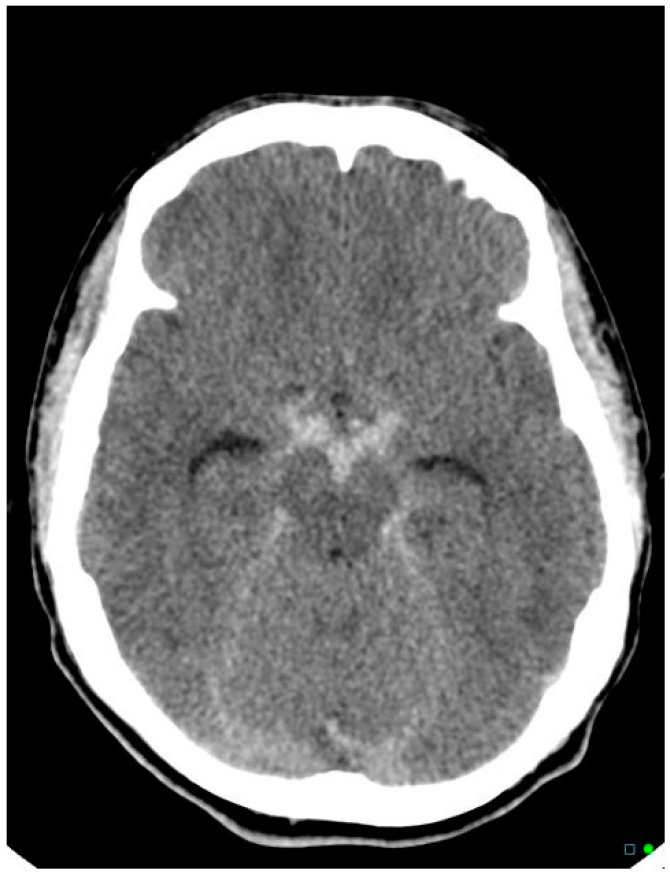
CT scan showed a thin layer of hyperdense subarachnoid blood encircling the upper brainstem, typical of a peri mesencephalic SAH. The 44-year-old patient presented with a GCS of 15 following a sudden-onset headache. CT angiography was normal.

**Figure 4:**
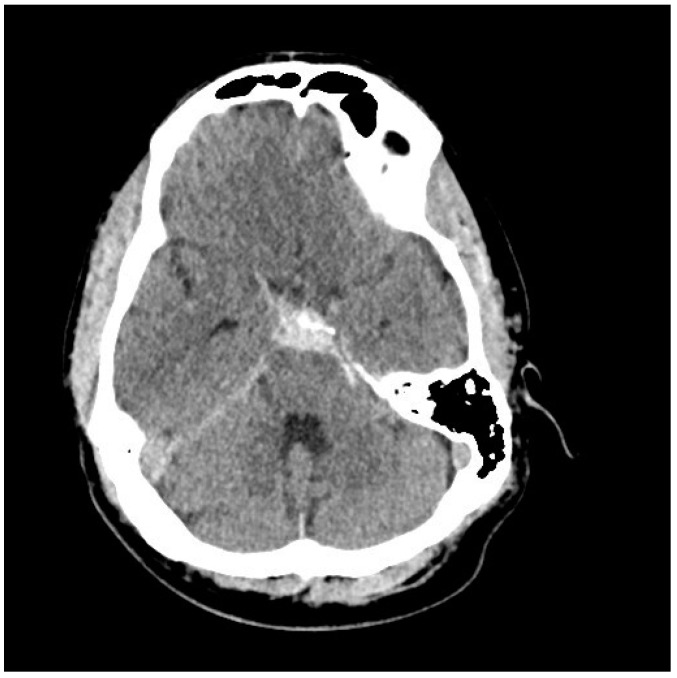
CT scan showing focal hyperdense subarachnoid clots near the midbrain and tentorial incisura, typical of a peri mesencephalic SAH. The 51-year-old patient presented with a GCS of 15 following a thunderclap headache. CT angiography was normal.

As mentioned, non-aneurysmal peri mesencephalic SAHs result in a quite characteristic pattern of SAH concentrated in the ambient cisterns around the midbrain and upper pons, and when typical in appearance, found in an alert patient, along with a normal CT angiogram, catheter angiography can be avoided [16] (Figure 3 and Figure 4). If bleeding is more extensive, involving the Sylvian fissures, for example, catheter angiography is prudent. Peri mesencephalic hemorrhages are most likely venous in origin and low volume; they usually clear rapidly, and acute hydrocephalus, vasospasm, and recurrent bleeding are all quite uncommon. While we have encountered transient hydrocephalus requiring EVD insertion, in our experience, this condition has never been complicated by symptomatic vasospasm or rebleeding.

Magnetic resonance imaging, especially the gradient echo sequence, can detect subarachnoid blood, and while not the investigation of choice for suspected aneurysm rupture, it might be performed first in patients presenting in a delayed fashion without a clear history of a thunderclap headache [17].

A rare cause of thunderclap headache is a condition known as “reversible cerebral vasoconstriction syndrome”, sometimes associated with childbirth or vasoactive drug use, and where small convexity SAHs are occasionally seen on CT scanning [18].

Catheter-based cerebral angiography is indicated for equivocal findings on CT angiography, such as a suspected but not clearly defined (usually small) cerebral aneurysm, a suspected dural AV fistula based on CT and CT angiography findings, and any large volume SAHs inconsistent with a peri-mesencephalic SAH, especially when the blood is thickest in the posterior fossa and therefore consistent with a spontaneous vertebral artery dissection.

Catheter-based angiography will, of course, precede endovascular aneurysm repair but is not necessary for most patients if aneurysm clipping is the chosen method of aneurysm repair.

## 6. Immediate Management of aSAH

Immediate treatment of aSAH is aggressive pain and blood pressure control, using intravenous narcotics and antihypertensives in good-grade patients, and airway control in poor-grade patients along with EVD insertion. If aneurysm repair is going to be delayed several hours for any reason (such as patient transport or the need to assemble a treatment team), the antifibrinolytic agent tranexamic acid should be administered to reduce the risk of early rebleeding (either 1 g as in the original study or 10 mg/kg intravenously) [19]. As will be discussed below, oral nimodipine (a calcium antagonist) is an indicated treatment for aSAH but not an immediate priority; we begin administration after aneurysm repair.

## 7. Aneurysm Repair

Elderly and infirm patients in poor neurological conditions are seldom appropriate patients for aneurysm ablation. High-grade patients, in general, particularly those who remain in grades 4 and 5 despite EVD insertion, need careful consideration by the aneurysm treatment team before proceeding to aneurysm repair. Younger patients who are comatose but with normal pupillary function and no vital brain destruction visible on CT scanning can make meaningful neurological recoveries after aSAH [20], so we believe securing their ruptured aneurysms is indicated.

Aneurysm repair should be carried out as soon as possible after presentation, the same day if possible, but after a late-day presentation and diagnosis, stabilization (discussed below), and repair the next day when the treatment team can be assembled is appropriate.

The options for aneurysm repair are either endovascular (typically coiling but, in some cases, detachment of recently introduced intrasaccular flow disruption devices) or microsurgical clipping. The sometimes-difficult choice is made based on the patient’s condition, age, aneurysm location, size, and neck width.

Endovascular treatment is favored for poor grade and older patients, especially when the aneurysm has a small neck favorable for coiling. However, there is still an important role for aneurysm clipping, especially in younger patients and for large and wide neck aneurysms which are more difficult to effectively treat with endovascular methods. Surgery is also required for patients with large intracerebral hematomas requiring evacuation due to mass effects.

The treatment choice is best made collaboratively by the entire treatment team, including the neurosurgeon, neuro-interventionalist (who is either a neuroradiologist but could also be a neurosurgeon trained in endovascular intervention), and neuro-intensive care physician. A randomized trial continues to study patients with fewer choices [21].

## 8. Complications of aSAH and Their Management

### 8.1. Vasospasm

Cerebral vasospasm is a prolonged, sometimes severe, but ultimately reversible narrowing of the cerebral arteries that begins days after aSAH. The risk for vasospasm depends mainly on the thickness of blood clots in the subarachnoid space and ventricles (a high Fisher grade discussed above).

Angiographic vasospasm is arterial narrowing seen on vascular imaging; it begins several days after SAH and peaks in severity about 1 week later. Clinical or symptomatic vasospasm narrowing causes cerebral ischemia with corresponding symptoms and signs and is also referred to as delayed cerebral ischemia (DCI).

Progression to DCI depends most importantly on the degree of arterial narrowing (mild, moderate, or severe) and its distribution (focal or diffuse). Vasospasm affects only the intradural arteries and primarily, but not exclusively, the large arteries and arterioles located on the brain’s surface, encased by subarachnoid blood.

The delayed onset and relative predictability of vasospasm provide a unique therapeutic window of opportunity not found in other types of ischemic stroke. Over the past several decades, improvements in our ability to detect and manage vasospasm have led to a major decline in patient morbidity and mortality from vasospasm, but it remains one of the important determinants of outcomes following aSAH.

Aneurysmal SAH management begins with maintaining systemic euvolemia and normal blood and intracranial pressures; “prophylactic hypervolemia” is harmful and contraindicated [22]. The calcium channel antagonist nimodipine remains the only proven pharmacological agent for vasospasm prevention and remains the standard treatment for aSAH, although its mechanism of action is unclear, and its actual impact on patient outcome is modest at best [23]. Nimodipine is administered orally or via nasogastric tube, 60 mg every 4 h, and continued for 3 weeks (for patients requiring that length of hospitalization). Nimodipine can cause temporary depression of blood pressure, in which case the dose can be halved, and the drug should be discontinued if it interferes with the treatment of symptomatic vasospasm with induced hypertension, discussed below. Nimodipine is not indicated for non-aneurysmal SAH and can be discontinued before 21 days if a patient is ready for discharge.

Although not conclusive, there is some evidence that statin therapy, such as pravastatin 40 md/d, can reduce the incidence of ischemic cerebrovascular events following aSAH, so given that adverse effects are few, some centers use them routinely [24]. However, we do not.

Other agents studied for vasospasm or ischemia prevention, such as intravenous magnesium and endothelin antagonists, are ineffective [23,25]. There is also evidence that prolonged lumbar drainage of CSF might reduce the risk of vasospasm development, although it is not indicated if the patient has an EVD in place [23].

Detection and diagnosis of vasospasm are assisted by regular bedside neurological examinations, serial transcranial Doppler testing, and, in appropriate patients, cerebral perfusion studies and vascular imaging [24]. Continuous electroencephalography (EEG) has been used in some centers and might be especially helpful in monitoring unresponsive high-grade aSAH patients, with several EEG features indicative of ischemia, such as a decreasing alpha-to-delta power ratio [23,25]. More invasive parenchymal probes measuring tissue oxygenation, lactate/pyruvate ratio, and glutamate to detect cerebral ischemia are more experimental in nature, technically demanding, and they sample and therefore monitor only a small volume of brain tissue [23,25], an important drawback.

The onset of DCI is subtle but progressive. Development of an arm drift or dysphasia signals internal carotid artery (ICA) and/or MCA vasospasm, while somnolence and abulia indicate possible anterior cerebral artery (ACA) spasm. Transcranial Doppler velocities trending up over several days to over 150 cm/second are concerning, and even in patients without a new deficit, should prompt a CT-angiogram. Poor grade and/or sedated patients where a neurological exam is difficult or impossible should routinely undergo at least 2 CT-angiograms looking for vasospasm during the vasospastic interval, days 5 to 12 post-SAH.

**Figure 5:**
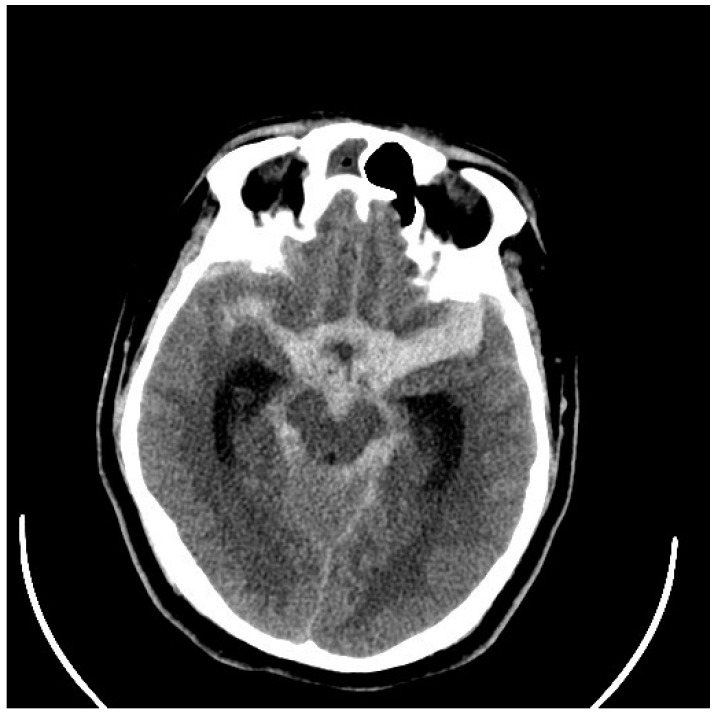
CT scan through the basal subarachnoid cisterns showing thick, hyperdense clots in a 31-year-old man who was drowsy and confused after a sudden onset headache with vomiting (Hunt and Hess and WFNS grade III). Note the thickest clot is in the left Sylvian fissure surrounding the left MCA (The following figures are all from the same patient).

**Figure 6:**
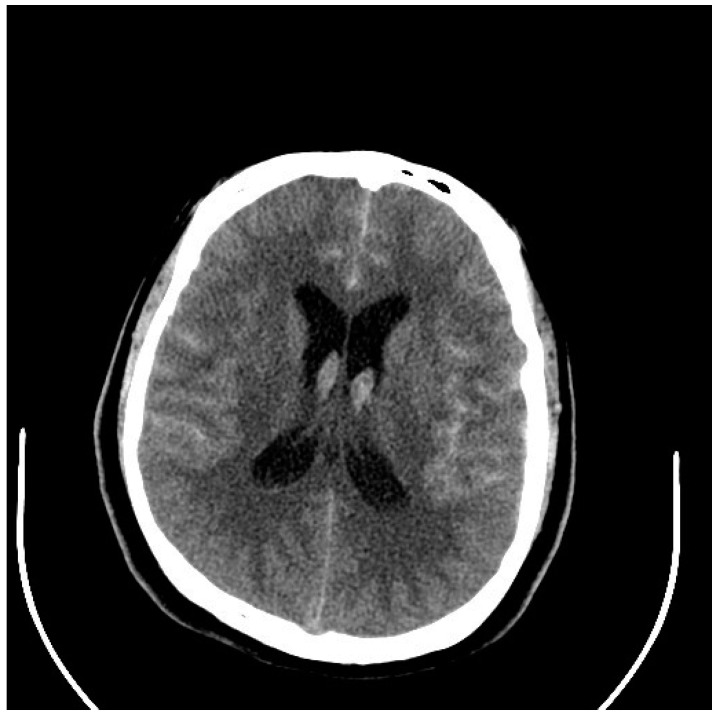
CT scan through the lateral ventricles showing intraventricular blood, indicating a Fisher grade 4 aSAH associated with a high risk of delayed cerebral vasospasm. An EVD was inserted.

**Figure 7:**
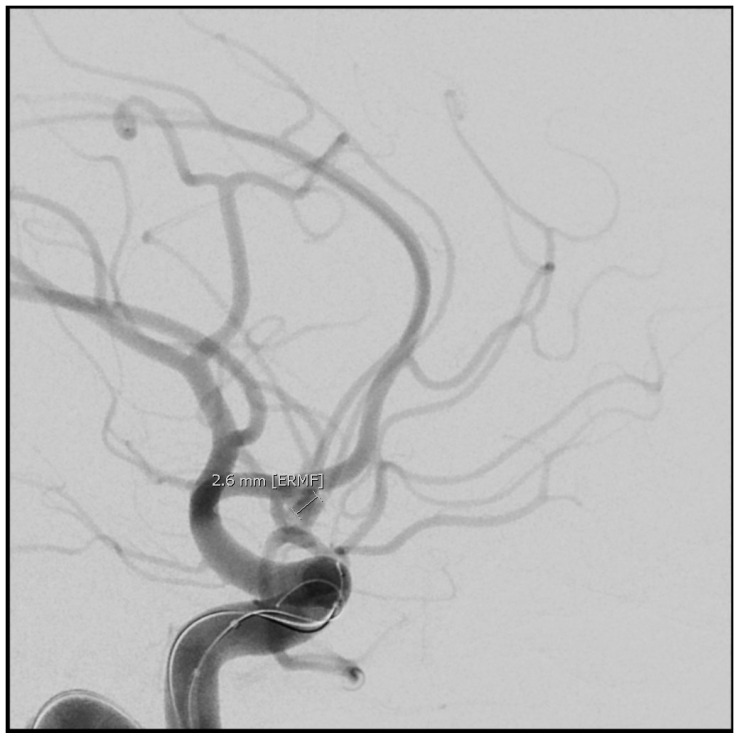
Catheter angiography immediately prior to coiling of a small anterior communicating aneurysm, just several millimeters in diameter. Note the normal caliber of the blood vessels to be compared with the next series of figures.

**Figure 8:**
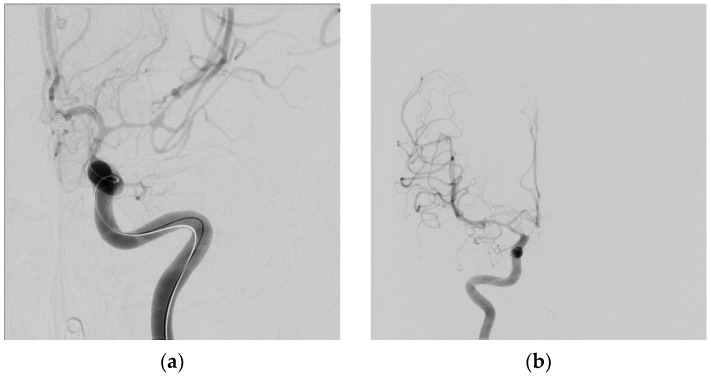
Seven days later, the patient became more lethargic with a new right-sided weakness. Velocities in the middle cerebral arteries on transcranial Doppler exceed 200 cm/s, indicating significant vasospasm. Hypertension was induced (see text), and after a CT ruled out cerebral infarction, a following catheter angiogram showed severe left MCA narrowing (**a**), where the thickest subarachnoid clot was located on the initial CT, and moderate vasospasm of the right MCA (**b**).

**Figure 9:**
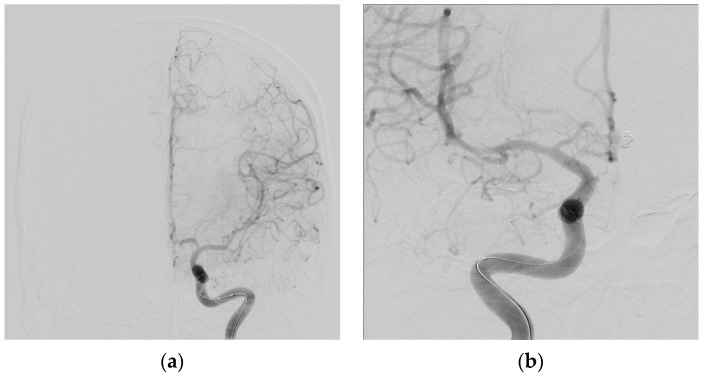
Post-angioplasty showing excellent reversal of the left MCA and proximal ACA vasospasm (**a**) the right MCA (**b**). All vasospastic vessels that could be reached with the balloon catheter were treated). The patient’s clinical condition improved and remained stable until full recovery.

**Figure 10:**
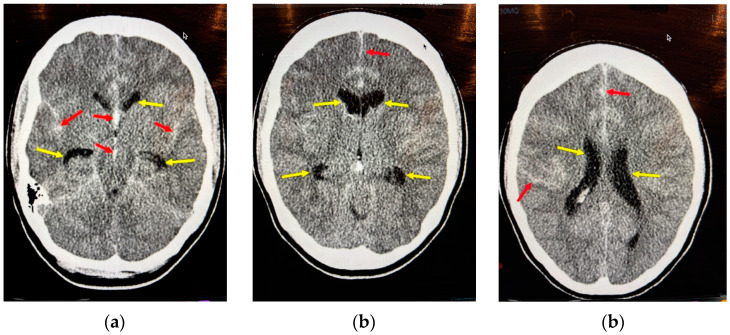
An example of acute hydrocephalus following aSAH secondary to a ruptured ACommA in a clinical aSAH grade 3 28-year-old woman. The red arrows point to subarachnoid blood in the cisterns, sulci, and interhemispheric fissure, and the yellow arrows point to prominent ventricles. This degree of hydrocephalus, while not florid, was clear with visible temporal horns (normally not visible in young adults) (**a**), and “rounding” of the frontal horns of the lateral ventricles (**b**). Combined with the flattening of the cerebral sulci (**c**), this CT scan is indicative of dangerously raised intracranial pressure, especially in a young patient requiring early EVD insertion.

Symptomatic vasospasm in examinable patients should be treated immediately with induced hypertension. The vasopressor can be either phenylephrine, norepinephrine, or a combination of both. Inotropic agents such as dobutamine, dopamine, and milrinone have also been used to treat vasospasm, but a prompt blood pressure response often requires a pressor. The key aspect of treatment, if it is to be successful in reversing ischemia, is prompt elevation of blood pressure. If deficits are not reversed within an hour after a target blood pressure (i.e., 200 mmHg) is reached, endovascular therapy (balloon angioplasty) to mechanically dilate the vasospastic arteries, improve blood flow and prevent cerebral infarction is indicated, providing CT scanning has ruled out either a new hemorrhage or an established infarct [23]. When faced with a patient with severe angiographic vasospasm where a prolonged period of vasospressor treatment to support perfusion is anticipated, our approach is to move directly to balloon angioplasty, treating all affected vessels (Figure 5, Figure 6, Figure 7, Figure 8, Figure 9 and Figure 10). Moderate or severe vasospasm detected on angiography in patients who cannot be properly assessed by clinical examination (either because of sedation or preexisting deficits) should be considered for balloon angioplasty as well, using clinical judgment on timing, but as soon as possible for severe vasospasm (Table 3).

If a patient fails to improve with hypertension and/or balloon angioplasty, it is probable that they have an established cerebral infarct or that the delayed onset deterioration is from another cause. This might be a blood gas, glucose or electrolyte disturbance, or an infectious process somewhere. It is important to remember that any existing neurological deficit due to brain injury is caused by rupture or aneurysm repair, for example, exacerbated by any of these systemic disturbances, thereby mimicking DCI. It has long been recognized that in aSAH studies, there has been a tendency to over-assign all types of delayed onset worsening to “vasospasm” when it was due to other causes.

### 8.2. Acute Hydrocephalus

Roughly one-third of aSAH patients develop acute hydrocephalus due to blockage of subarachnoid pathways and arachnoid granulations by hematoma and erythrocytes (external hydrocephalus) or by ventricular hematomas (internal hydrocephalus) [26]. Hydrocephalus is associated with larger volume SAHs, intraventricular hematomas, poorer clinical grade, and ACommAs, and it is a life-threatening condition (Figure 10). External ventricular drains are indicated for all patients with notable ventricular dilatation regardless of grade, especially in young patients with little intracranial compliance, and for grade IV and V patients because, as mentioned already, ventricular drainage may improve their neurological status.

The timing of EVD insertion must consider the clinical circumstances prior to a coiling procedure (insertion usually performed in the intensive care unit) and either preoperatively again in the intensive care unit or intraoperatively immediately prior to a clipping procedure.

There is some evidence that low serum magnesium (Mg) (Mg being important for the biological activity of clotting factor IX) is associated with catheter tract bleeding following EVD placement. This finding has suggested a possible therapeutic role of magnesium sulfate (MgSO_4_, 2 gm IV) prior to drain insertion [27]. There is more robust evidence for using either antibiotic-impregnated catheters or prophylactic intravenous antibiotics prior to drain insertion [28].

The insertion of an EVD is a neurosurgical procedure. The location of either the twist drill or burr hole through the frontal bone is just in front of the coronal suture on the right side, three cm lateral to the midline. The neurosurgeon or neurosurgical trainee performing the procedure must know the external landmarks that localize the coronal suture: the coronal suture is beneath the line connecting points on the scalp drawn one thumb breadth behind the frontal process of the zygomatic bone or two finger breadths anterior to the two external auditory meati. The catheter is aimed towards the midline and inserted no deeper than 6 cm from the skin surface. Failed or poor catheter placements are prevalent when care is not taken with each step.

High vasospasm-risk patients (discussed above) require a longer drainage period, often 10 to 14 days, whereas low-risk patients with less clot burden will have their drains removed sooner. There are two weaning strategies: a gradual, continuous approach increasing the resistance over several days versus a rapid “abrupt” approach of clamping the drain and reopening only in the event of clinical deterioration and/or preset intracranial pressure (ICP) criteria [29].

Without any clear evidence of the superiority of one method over the other, we follow a “hybrid” approach where the goal is to minimize the number of conversions to ventriculoperitoneal (VP) shunts. Weaning is carried out continuously over several days until the daily drainage is less than 150 cc in 24 h against 20 cm of H_2_0 (the daily production of CSF is approximately 500 cc). At this point, the drain is clamped and reopened for 15 min (against 20 cm H_2_0) only in the event of unequivocal neurological decline and *not* for a complaint of headache alone or any arbitrary ICP measurement, providing the patient has a normal GCS. If the CT scan the following day rules out hydrocephalus, then the EVD is removed. In our experience, approximately 20% of patients will subsequently require a VP shunt.

Intraventricular fibrinolysis using recombinant tissue plasminogen activator (rt-PA) has been shown to rapidly clear large aneurysmal IVHs and can be considered both for assistance in ICP management and maintenance of ventricular catheter patency [30]. The dose we use is 4 mg in 4 cc of saline, administered via the EVD directly; the EVD is then closed and opened at alternate hours.

### 8.3. Raised Intracranial Pressure

Hydrocephalus and large intracerebral or intraventricular hematomas have been discussed. Aneurysms located at the AComm can cause large frontal hematomas and MCAAs large temporal lobe or frontotemporal hematomas, requiring at least partial evacuation due to ICP concerns. When confronted with these patients such as these, surgeons should perform large craniectomies, either bifrontal (ACommAs) or frontotemporal (MCAAs), anticipating progressive intracranial hypertension.

Poor-grade patients with more severe SAHs can also suffer diffuse brain swelling, resulting in progressively worse intracranial hypertension.

Elevating the head of the bed and ensuring the neck is free from constriction from endotracheal tube tape is, of course, important. Patients are sedated and sometimes paralyzed, often making neurological assessments limited to pupil checks. Intravenous propofol is commonly used for sedation, preferred because it allows rapid neurological assessments once the infusion is held, and it can be paired with fentanyl if analgesia is required for any reason.

With a goal of maintaining the cerebral perfusion pressure (CPP—mean arterial blood pressure minus the ICP) of at least 60 mmHg, hyperosmolar treatment (HOT) with hypertonic saline (HTS) and/or intermittent mannitol infusions are used. Hypertonic saline can be given either as intermittent boluses (9%) or continuous infusion (3%). There is no clear evidence of one treatment being superior to another, but all types of hyperosmolar treatments require close monitoring of serum sodium concentrations, with acute renal injury a serious threat [31].

Another grave, sometimes ultimately fatal development after aSAH is a large territorial infarct due either to a complication of aneurysm repair or vasospasm, as discussed above. Bifrontal or hemi cranial decompressive craniectomies are considered in selected patients as a desperate life-saving measure in the face of intractable intracranial hypertension. With more than three decades of treating patients with aSAH, we have had very limited success with these quite aggressive interventions under these circumstances.

### 8.4. Seizures

It is important to appreciate that episodes of aneurysm rebleeding can closely resemble a generalized seizure, so after such an event prior to aneurysm repair, a repeat CT scan should be performed to rule out this possibility.

True epileptic seizures complicate roughly 10% of aneurysm ruptures, with poor aneurysm grade, cerebral infarction, hemorrhage, and edema all increasing the risk [31,32]. Routine antiseizure prophylaxis is not recommended after aSAH. Antiepileptic agents should be administered only after a documented epileptic convulsion or nonconvulsive seizures are detected with electroencephalography, and phenytoin should be avoided; levetiracetam is a good alternative [33,34].

### 8.5. Electrolyte, Hemoglobin, and General Measures

Patients with aSAH can develop excessive natriuresis, known as “cerebral salt wasting”, due to elevations in brain natriuretic peptide secretions. This can lead to hyponatremia and an increased risk of delayed cerebral ischemia. An alternative cause of hyponatremia, the syndrome of inappropriate antidiuretic hormone elevation (SIADH), is comparatively rare following aSAH. Hyponatremia after aSAH should aim to reverse cerebral salt wasting with 3% hypertonic saline infusions combined with fludrocortisone at a measurement of 0.1 or 0.2 mg/d. Fluid restriction (used to treat SIADH) and resulting hypovolemia are contraindicated after aSAH.

Blood transfusions are recommended if the patient’s hemoglobin falls below 8 g/dL [35].

Optimal ventilation and oxygenation, fever prevention, close glucose control, nutrition, and attention to all electrolytes in addition to sodium are all, of course, important in the medical management of aSAH, no different from any other critically ill patient.

Lastly, it is important to emphasize that the care of aSAH is a multidisciplinary task that includes neurosurgeons and interventional neuroradiologists, but equally important are emergency and intensive care physicians, nurses, therapists, and technicians knowledgeable about the condition, following a patient-centered approach in improving outcomes.

## Figures and Tables

**Table 1:** Cumulative risk table for unruptured intracranial aneurysms.

Age at Diagnosis	Remaining Years to Age 80	Risk of Hemorrhage 1% per Year	Risk of Hemorrhage 2% per Year	Risk of Hemorrhage 3% per Year
35	45	35	60	75
45	35	29	51	66
55	25	22	40	53
65	15	18	26	37
75	5	10	10	14

**Table 2:** Grading scales for aSAH outcome prediction (prognosis worse for higher Hunt/Hess, WFNS “grades” and lower GCS “scores”) and symptomatic vasospasm prediction (greater symptomatic vasospasm risk with higher Fisher grades).

Hunt and Hess SAH Grading Scale [8]	Glasgow Coma Scale (GCS, out of 15)	WFNS SAH Grading Scale (Modified) [9]	Fisher SAH Scale (Modified) for Vasospasm Prediction [10]
Grade 1: Headache Grade 2: Severe headache but alert Grade 3: Drowsy Grade 4: Coma Grade 5: Coma with abnormal brainstem findings	Eye opening: 4. spontaneous 3. to speech 2. to pain 1. none Speech: 5. alert 4. confused 3. inappropriate 2. incomprehensive 1. none Motor response: 6. normal 5. localizing 4. withdrawal 3. decorticate 2. decerebrate 1. none	1. GCS 15 2. GCS 13–14, no focal deficit or meningismus 3. GCS 13–14, mild deficit or meningismus present 4. GCS 7–12 5. GCS <7	Thin SAH, no IVH (low VSP risk) Thick SAH, no IVH (intermediate VSP risk) Thin SAH, IVH present (intermediate VSP risk) Thick SAH and IVH present (high VSP risk) IVH = intraventricular hemorrhage thin SAH is <1 mm VSP = symptomatic vasospasm

**Table 3:** Stepwise management of symptomatic vasospasm.

Five-Step Management of Symptomatic Vasospasm Complicating aSAH
Rule out other cause(s) of neurological deterioration (including hypoxia, hyponatremia, and pyrexia). Rule out new intracranial hemorrhage or established cerebral infarction with immediate CT scanning. Ensure and maintain euvolemia. Prompt the inducement of hypertension (minimum 200 mmHg systolic) with vasopressor(s). If deficits fail to fully reverse within one hour, prompt endovascular angioplasty. Balloon angioplasty should be considered early for all patients found to have severe angiographic vasospasm, treating all affected vessels.
